# Marine phytoplankton community data and corresponding environmental properties from eastern Norway, 1896–2020

**DOI:** 10.1038/s41597-022-01869-3

**Published:** 2022-12-14

**Authors:** Elisabeth Lundsør, Evy Rigmor Lømsland, Torbjørn Martin Johnsen, Anette Engesmo, Andrew Luke King, Andrè Staalstrøm, Marit Norli, Jan Magnusson, Kai Sørensen, Bente Edvardsen, Wenche Eikrem

**Affiliations:** 1 Department of Biosciences, University of Oslo, PO Box 1066 Blindern, 0316 Oslo, Norway; 2 Norconsult AS, PO Box 626, 1303 Sandvika, Norway; 3 Norwegian Institute for Water Research (NIVA), Gaustadalléen 21, 0349 Oslo, Norway

**Keywords:** Marine biology, Ecosystem ecology, Biodiversity

## Abstract

Time series are essential for studying the long-term effects of human impact and climatic changes on the natural environment. Although data exist, no long-term phytoplankton dataset for the Norwegian coastal area has been compiled and made publicly available in a standardised format. Here we report on a compilation of phytoplankton data from inner Oslofjorden going back more than a century. The database contains 605 sampling events from 1896 to 2020, and environmental data has also been provided when available. Although the sampling frequency has varied over time, the high taxonomic quality and relatively similar methodology make it very useful. For the last 15 years (2006–2020), the sampling frequency has been almost monthly throughout the year. This dataset can be used for time series analysis to understand community structure and changes over time. It can also be used to study common taxa’ responses to environmental variables and changes, seasonal or annual species diversity and be useful for developing ecological indicators.

## Background & Summary

The inner Oslofjorden Phytoplankton Database is a comprehensive database containing quantitative phytoplankton cell counts, associated metadata and available environmental data. The primary source for the database is the monitoring programme for inner Oslofjorden conducted with varying yearly frequencies from 1973 until today, mainly by the Norwegian Institute for Water Research (NIVA). From 2006 to 2020, the sampling frequency was approximately monthly. Secondly, the database is also supplied with data from various projects from 1896 to 1976 conducted by researchers from the University of Oslo (UiO).

The database is most comprehensive for the station, Dk1 (S1) in Vestfjorden, but also includes some less complete data from other stations in the inner Oslofjorden. The database consists of 605 sampling events resulting in 22635 phytoplankton taxon records. The database can be accessed from 10.15468/gugesq^1^ and provides high-quality phytoplankton abundance data. The species taxonomy is updated, and the count values are quality checked and standardised. Metadata, like sampling date, sampling location, sampling depth and methodology, is provided and standardised. Additionally, associated abiotic data is available for most samples, and biomass data is available from 1994 to 2020, with some exceptions. The data set allows for analyses of long-term temporal trends in phytoplankton community structure, including changes in phytoplankton phenology and seasonality.

The inner Oslofjorden is a recipient for the city of Oslo (the capital of Norway), and eutrophication’s impact on the phytoplankton community has been documented through surveys from the early 1900s^2^. Therefore, a survey was carried out by the UiO in 1933–34, which showed that the seasonal patterns of phytoplankton were very different in the inner and outer parts of the fjord caused by higher nutrient loads in the inner fjord^3^. Another extensive study by the UiO in 1962–65 documented that the upper water column was heavily eutrophic, and nutrient supply from land-based activities was one of the primary sources causing this problem^4^. Consequently, annual monitoring surveys were initiated in 1973 and are still ongoing^5,6^.

The inner Oslofjorden is a sill fjord of 190 km^2^ in size. The fjord is connected to the more open outer Oslofjorden and Skagerrak through the narrow sound at Drøbak, where the sill is only 19.5 m deep. North of the Drøbak sill, more sills divide the fjord into several basins, such as Vestfjorden (basin depth 162 m), Bærumsbassenget (31 m), Bekkelagsbassenget (72 m), and Bunnefjorden (152 m). The bathymetry is a constraint to efficient deep-water renewal^7,8^. Deep water is renewed only every 3–5 years in the innermost parts (Bunnefjorden) but yearly in the outer parts (Vestfjorden)^9^. The deep-water renewal also depends on the variation between the basins’ vertical diffusion, reducing the density in the deep water between exchanges^10^.

The limited water exchange makes the fjord particularly vulnerable to pollution. The high impact of sewage with nutrients and organic matter leads to high phytoplankton concentrations in surface waters and a high level of oxygen consumption in the deep water^7^. Sewage treatment started primarily in 1963 and has reduced eutrophication’s impacts.

Inner Oslofjorden is a relatively sheltered area with calm weather, warm summers, and cold winters, with dominating southerly winds in the summer and northerly winds during winter. Extended periods of strong northerly winds are favorable for water exchange when south-streaming surface water is replaced with north-streaming heavier and oxygen-rich deep water from the inner Skagerrak and outer Oslofjorden. The heavier (mainly higher salinity) water enters over sill depth at Drøbak and replaces the old (lighter) deep water. Thus, the inner fjord’s deep water’s oxygen concentration increases^11^.

Rivers, waterways, and land runoff supply bioavailable phosphate to the fjord, but the contribution from sewage plants, especially overflow runoff, can also be substantial. However, the major delivery of organic substances is through the discharge from the sewage plants^12^.

In inner Oslofjorden, the water column is stratified all year round. However, stability varies with season, with a minimum in winter and gradually increasing during early spring towards maximum stability in the summer. In the autumn, a gradual stability decline occurs, as in northern waters in general.

## Methods

### Sampling strategies and data

The inner Oslofjorden phytoplankton dataset is a compilation of data mostly assembled from the monitoring program, financed since 1978 by a cooperation between the municipalities around the fjord, united in the counsel for technical water and sewage cooperation called “Fagrådet for Vann- og avløpsteknisk samarbeid i Indre Oslofjord”. The monitoring program started in 1973 and is ongoing. The program has sampled environmental parameters and chlorophyll since 1973, but for the first 25 years, phytoplankton data is only reported for the years 1973, 1974, 1988/9, 1990, 1994 and 1995. Since 1998, yearly sampling has been conducted, and from 2006 to 2019, the sampling frequency was approximately monthly. In addition, we have compiled research and monitoring data from researchers at the University of Oslo from 1896 and 1916, 1933–34 and 1962–1965.

The records from 1896 and 1897 were collected using zoo-plankton net^13^. The phytoplankton collection in 1916–1917 used buckets or Nansen flasks for sampling. From 1933 to 1984, phytoplankton samples were collected using Nansen bottles and then from 1985–2020 with Niskin bottles from research vessels. The exception is the period from 2006 to 2018 when samples were also collected with FerryBox- equipped ships of opportunity^14^ with refrigerated autosamplers (Table 2).

Since the 1990s, quantitative phytoplankton samples have mostly been preserved in Lugol’s solution, except for spring and autumn samples in the period 1990–2000 that were preserved in formalin. The records from 1896, 1897 and 1916 were preserved in ethanol, and between 1933 and 1990, samples were preserved in formalin. Sampling strategies and methods are listed in Table 2.

The records from 1896 and 1897 were quantified by weight, and taxon abundance is categorised as “rare” (r), **“rather common”** (+), “common” (c) and “very common” (cc)^13^. In 1916 and 1917, Grans filtration method^15^ was used, and the number was given in cell counts per litre. From 1916 to 1993, the data is reported only as phytoplankton abundance (N, number of cells per litre). For most years after 1994, the dataset includes both abundance and biomass (μg C per litre), except for 2003, 2004, 2017 and 2018. Phytoplankton was identified and quantified using the sedimentation method of Utermöhl (1958)^16^. Biovolume for each species is calculated according to HELCOM 2006^17^ and converted to biomass (μg C) following Menden-Deuer & Lessards (2000)^18^.

### Data inventory

The inner Oslofjorden Phytoplankton dataset was compiled in 2020, comprising quantitative phytoplankton cell counts from inner Oslofjorden since 1896. Previously, parts of the data have been available as handwritten or printed tables in reports and published sources^19–21^ (Fig. 2). All sources are digitally available from the University of Oslo Library, the website for “Fagrådet” (http://www.indre-oslofjord.no/) or the NIVA online report database (https://www.niva.no/rapporter). Data from 1994 and onwards have been accessed digitally from the NIVA’s databases. They are also available from client reports from the monitoring project for inner Oslofjorden from the online sites listed above.

The first known, published investigation of hydrography and plankton in the upper water column of the inner Oslofjorden was by Hjort & Gran (1900)^13^. Samples were collected during a hydrographical and biological investigation covering both the Skagerrak and Oslofjorden. There is only one sampling event from Steilene (Dk 1), but some phytoplankton data were obtained at Drøbak, just south of the shallow sill separating the inner and outer Oslofjorden, from winter 1896 to autumn 1897. Twenty years later, Gran and Gaarder (1927)^22^ conducted a study that included culture experiments at Drøbak field station (at the border between the inner and outer Oslofjorden) in March - April 1916 and August - September 1917. A higher frequency investigation was carried out from June 1933 to May 1934, covering 12 stations in inner and outer Oslofjorden where phytoplankton was analysed by microscopic examination^23^. The extensive program (the Oslofjord Project) conducted from 1962–1964 covered many parameters, and we have extracted the data for phytoplankton. From 1973 and onward, the research vessel-based monitoring program was financed by the municipalities around the fjord, and since 2006 NIVA has supplemented the monitoring program using FerryBox ships of opportunity. Samples from 4 m depth were collected using a refrigerated autosampler system (Teledyne ISCO) connected to a FerryBox system on M/S *Color Festival* and M/S *Color Fantasy* through cooperation between NIVA and Color Line A/S. Since 2018, the FerryBox has been part of the Norwegian Ships of Opportunity Program research infrastructure funded by the Research Council of Norway.

The indicated depth of 3.5–4 m is an estimated average, as the actual sampling depth depends on shipload and sea conditions.

Several other research projects have sampled from inner Oslofjorden between 1886 and 2000 with different aims. Data from relevant projects reporting on the whole phytoplankton community have also been included in this database.

### Data compilation

The data already digitalised were compiled from MS Excel files, and other data were manually entered into the standard format in MS Excel files. All collected data were then integrated into one MS Excel database, and this file was used for upload into GBIF. Data can be downloaded from GBIF in different formats and be linked together by the measurementsorfacts table.

### Quality control and standardisation

After compilation, the data were checked for errors that could occur during manual digitalisation or just the compilation process. Duplicates and zero values were removed (Fig. 2). The major quantitative unit is phytoplankton abundance in cells per litre. Due to varying scopes of sampling and the development of gear and instruments, the number of species identified may vary between projects. Some of the earliest records were registered as “present”, indicating the amount in comments.

Metadata, such as geographical reference, depth and methodology accessed from papers and reports, were accessible from the data source. When data was accessed from the NIVA internal databases, the metadata information was provided by the database owners/researchers.

### Taxonomy

The taxonomy of microalgae is in constant revision as new knowledge and techniques for identification are developing. Several historical species names recorded in this database are synonyms of accepted names in 2021. We have used the original names in our database and matched them to accepted names and Aphia ID using the taxon match tool available in the open-access reference system; World Register of Marine Species (Worms)^24^. The taxon match was conducted in March 2021.

The nomenclature in Worms is quality assured by a wide range of taxonomic specialists. The Aphia ID is a unique and stable identifier for each available name in the database^24^. We also cross-checked the last updated nomenclature in Algaebase^25^ (March 2022) to assign species to a valid taxon name. When Algaebase and Worms were not in accordance, Algaebase taxonomy was usually chosen except in the case of Class Bacillariophyceae.

Before matching the species list, the original species names were cleaned from spelling mistakes or just spelling mismatches like spaces, commas, etc. The original name is, however, left in one column in the database. For registrations where a species identification is uncertain, e.g. *Alexandrium* cf. *tamarense*, we used only *Alexandrium*. For registrations where the full name is uncertain, e.g. cf. *Alexandrium tamarense*, we used the name and Aphia ID for higher taxa, in this case, order. For others, e.g. “pennate diatoms” or “centric diatoms“, we used the name and Aphia ID for class. When names for, e.g. order and class were not recognised automatically by the matching tool in World Register of Marine Species (WoRMS), these were matched manually. Only very few records, mostly “cysts” and “unidentified monads”, could not be matched neither automatically nor manually but were assigned to general “protists” with affiliated ID.

## Data Records

The inner Oslofjorden Phytoplankton Database can be accessed from the Global Biodiversity Information Facility, 10.15468/gugesq^1^.

## Record types

In total, 22636 phytoplankton records are stored in the database. Quantitative units are phytoplankton abundance in cells (N) per litre. Many records from 1994 to 2020 also contain registrations of biomass in µg carbon (C) per litre.

Each data record is linked to its associated metadata, such as information about the sampling event, sampling depth, taxonomy, and data source.

### Temporal coverage

**Fig. 1:**
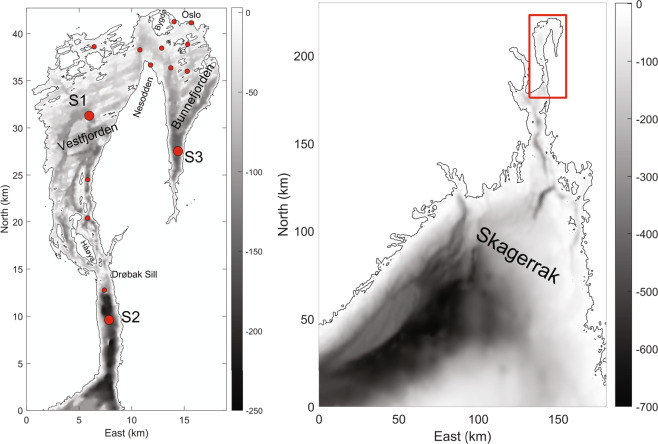
Locations of the 605 sampling events included in the database. Most of the data is from station S1. Stations S2 and S3 do also have large amounts of data. Additional sampling stations are indicated with smaller dots.

The database covers several sampling stations in the inner Oslofjorden, with the majority of samples (61%) from Dk1 (S1) (Fig. 1). Dk1 has been the most sampled station throughout all the years of sampling. Data are available for the years 1896–97, 1916–17, 1933–1934, 1939, 1948, 1957–58, 1962–65, 1972–1974, 1988–1990, 1994–1995 and 1998–2020 (Fig. 3). From 1998–2004 there were only samples during the summer months (May to Aug), but from 2006 to 2020, there was good seasonal coverage (Fig. 4).

**Fig. 2:**
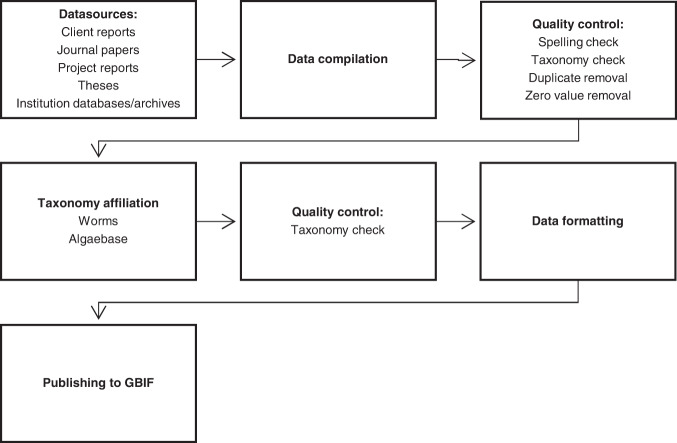
Workflow for compilation of the inner Oslofjorden phytoplankton database.

**Fig. 3:**
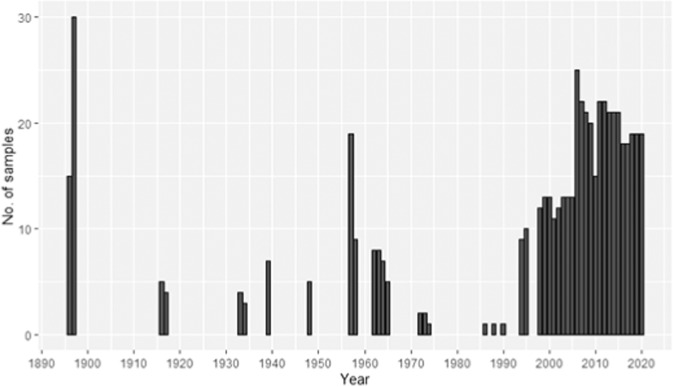
Amount of phytoplankton sampling events per year registered in the inner Oslofjorden phytoplankton database.

**Fig. 4:**
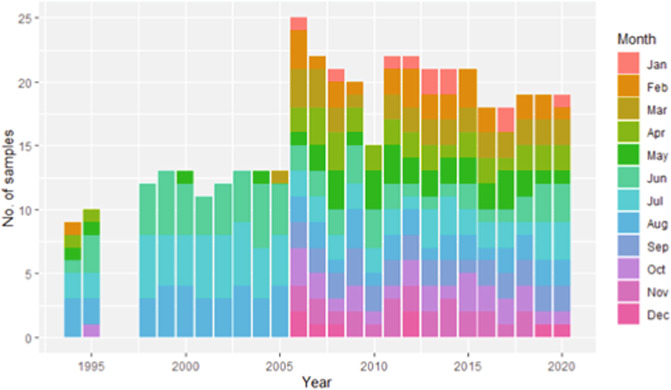
Amount of phytoplankton sampling events in the years 1994–2020 registered in the inner Oslofjorden phytoplankton database. The months sampled are indicated with colours; see the legend.

### Taxonomic coverage

The database contains 412 unique accepted taxa registrations of which 75% are identified to the genus or species level. These records are distributed among 17 classes. Approximately 9% of the registrations are “flagellates“, “monads” (non-flagellated unidentified cells) or other taxa not identified to class. The most counted classes in the dataset are Dinophyceae (dinoflagellates) and Bacillariophyceae (diatoms), representing 38% and 34%, respectively, of all records (Table 1).

**Table 1:** Frequency of observations distributed among taxonomic phytoplankton classes present in the inner Oslofjorden Phytoplankton Database.

Taxonomic Class	Number of observations	Percentage of observations
Dinophyceae	8500	37.55%
Bacillariophyceae	7798	34.45%
Cryptophyceae	1075	4.75%
Coccolithophyceae	1057	4.67%
Dictyochophyceae	490	2.16%
Pyramimonadophyceae	394	1.74%
Euglenoidea	301	1.33%
Chrysophyceae	298	1.32%
Litostomatea	222	0.98%
Cryptophyta incertae sedis	181	0.80%
Cyanophyceae	85	0.38%
Raphidophyceae	48	0.21%
Chlorophyceae	37	0.16%
Trebouxiophyceae	20	0.09%
Chlorodendrophyceae	14	0.06%
Mamiellophyceae	11	0.05%
Zygnematophyceae	1	0.004%
Unidentified	2092	0.004%

### Associated environmental data

When available, the associated environmental abiotic data measured during the same sampling events as the phytoplankton collection are also included. The records differ according to the scope of the project but contain, e.g., concentrations of nutrients, dissolved oxygen and chlorophyll *a*, temperature, and salinity. Water samples were collected for nutrients and chlorophyll *a* analyses using the same techniques as for collecting phytoplankton samples. Temperature, salinity, and dissolved oxygen concentration were measured using CTD and oxygen sensors either as part of CTD-rosette systems deployed on research vessels or FerryBox sensor systems on ships of opportunity. Like the phytoplankton data, these data are mainly analysed at the laboratories at the UiO or NIVA.

Changes in methods have however occurred over the years^26^. The data have been quality checked and referenced in time. Duplicates, outliers, and odd values have been removed. The environmental data can be either directly linked to the phytoplankton data via a common “Event” (Sample ID) or via a combination of sampling date, depth, and station.

## Technical Validation

Data of high-quality phytoplankton count data and its environmental data of several decades are recorded in our database. As the database is a compilation of data from several projects, some factors should be considered before use. Within the century-long period covered by the database, sampling, preservation, and taxonomic knowledge have improved (Table 2). The records from before 1920 are not directly comparable with the other as there are no individual cell counts before 1900 and different preservation and counting protocols have been used. From the 1930s and onwards, the same protocol for sampling and counting has been used (Table 2). Over the years, several researchers have performed taxonomic identification and cell counts recorded in this database. Although well-trained and with a quality assurance system in place, the human component in microscopic taxonomic determination can never be excluded altogether. Variable levels of taxonomic expertise and differing species delimitations practices, together with the fact that taxonomic skills will improve even during single’ taxonomists’ careers, is well-known^27^.

**Table 2:** Presentation of methodology for phytoplankton sampling, preservation and analysis over the timeframe covered by the database.

Sampling year	Sampling method	Preservation method	Analythical method
1896–97	Petersen self-closing vertical net	Ethanol, 70%	Weight of quantity
1916–17	Nansen flask or bucket	Ethanol, 70%	Grans filtration method
1933–34	Nansen water bottles	Formaldehyde, 2–4%	Utermöhl sedimentation^16^
1939	Nansen water bottles	Formaldehyde, 2–4%	Utermöhl sedimentation
1948	Nansen water bottles	Formaldehyde, 2–4%	Utermöhl sedimentation
1957–58	Nansen water bottles	Formaldehyde, 2–4%	Utermöhl sedimentation
1962–65	Nansen water bottles	Formaldehyde, 2–4%	Utermöhl sedimentation
1972–1974	Nansen water bottles	Formaldehyde, 2–4%	Utermöhl sedimentation
1988–1990	Niskin water bottles	Formaldehyde, 2–4%	Utermöhl sedimentation
1994–2005	Niskin water bottles	Lugol’s solution^16^, 1%	Utermöhl sedimentation
2006–2018	FerryBox autosampler	Lugol’s solution, 1%	Utermöhl sedimentation
2019–2020	Niskin water bottles	Lugol’s solution, 1%	Utermöhl sedimentation

Concentrations of fixatives are final concentrations. Lugol’s fixed samples are as a rule analysed within weeks, whereas formaldehyde fixed samples can be preserved for years.

However, all data present in the database were obtained in a few well-equipped laboratories at either the University of Oslo or NIVA, known for their high research standards and extensive expertise in phytoplankton identification and taxonomy. Therefore, the phytoplankton identifications and counts are generally solid.

This database represents marine phytoplankton records from a fjord environment with a long history of different types and levels of nutrient inputs. This database represents is the only Norwegian phytoplankton database that contains data going back more than a century. It also includes associated environmental data from the same sampling events when these have been available.

## Usage Notes

The database contains data on phytoplankton cell counts from more than a century of sampling and long periods with good seasonal coverage. Together with the associated environmental data this can be used for time series analysis to determine community structure and changes over time. It can also be used to study common taxa’s responses to environmental variables and changes, seasonal or annual diversity and be useful for developing ecological indicators.

The data can be analysed using various tools such as the open software R^28^.

## Data Availability

No specific code was generated for analysis of these data.

## References

[1] Lundsør E, Aebersold R (2022). GBIF.

[2] Braarud, T. Pollution effect upon the phytoplankton of the Oslofjord. *Int. Counsil Explor. Sea* 1–20 (1969).

[3] Braarud, T. *A Phytoplankton Survey of the Polluted Waters of Inner Oslo Fjord*. (Det Norske Vitenskapsakademi i Oslo, 1945).

[4] Munthe-Kaas H (1968). Surface pollution and light extinction in the Oslofjord. Helgoländer Wissenschaftliche Meeresuntersuchungen.

[5] Staalstrøm, A., Engesmo, A., Andersen, G. S. & Hjermann, D. Ø. *Undersøkelse av hydrografiske og biologiske forhold i Indre Oslofjord*. (Norwegian Institute for Water Research, 2020).

[6] Magnusson, J. & Källkvist, T. *Undersøkelse av de hydrografiske og biologiske forhold i indre Oslofjord. Overvåkningsprogram Årsrapport 1973*. (Norwegian Institute for Water Research, 1974).

[7] Staalstrøm, A. Tidally-induced turbulent mixing in a sill fjord. (University of Oslo, 2015).

[8] Magnusson, F. J. & Berge, J. A. Overvåking av Indre Oslofjord i 2014. 1–24 (Norwegian Institute for Water Research, 2015).

[9] Baalsrud, K. & Magnusson, J. *Indre Oslofjord; Natur og Miljø*. (Fagrådet for vann- og avløpsteknisk samarbeid i indre Oslofjord, 2002).

[10] Stigebrandt A (1976). Vertical Diffusion Driven by Internal Wawes in a Sill Fjord. J. Phys. Oceanogr..

[11] Gade HG (1968). Horizontal and vertical exchanges and diffusion in the water masses of the oslo fjord. Helgoländer Wissenschaftliche Meeresuntersuchungen.

[12] Vogelsang, C. *Strategi 2010. Samlet vurdering av resultatene fra modellsimuleringer med NIVAs fjordmodell og fra studiet av tilførsler av omsettelig organisk stoff fra renseanlegg og elver*. (Norwegian Institute for Water Research, 2011).

[13] Hjort J, Gran HH (1900). Hydrographic-Biological Investigations of The Skagerrak and the Christiania Fiord. Rep. Nor. Fish. Mar..

[14] Magnusson, J. *et al*. *Validation of methods for monitoring of coastal and open sea areas with satellites and sensors on ships of opportunity*. *REPORT SNO* 4710–2003 (2003).

[15] Gran HH (1912). Preservation of samples and quantitative determination of the plankton. ICES J. Mar. Sci..

[16] Utermöhl H (1958). Zur Vervollkommnung der quantitativen Phytoplankton-Methodik. SIL Commun. 1953–1996.

[17] Olenina, I. *et al*. *Biovolumes and size-classes of phytoplankton in the Baltic Sea*. *HELCOM Balt.Sea Environ. Proc*. (2006).

[18] Menden-Deuer S, Lessard EJ (2000). Carbon to volume relationships for dinoflagellates, diatoms, and other protist plankton. Limnol. Oceanogr..

[19] Braarud, T. & Nygaard, I. *Fytoplankton. Delrapport nr 4. Oslofjorden og dens Forurensningsproblemer*. (University of Oslo, 1967).

[20] Magnusson, J., Källkvist, T., Pedersen, A. & Tangen, K. *Overvåking av forurensningssituasjonen i Indre Oslofjord 1982*. (Norwegian Institute for Water Research, 1984).

[21] Magnusson, J., Källkvist, T. & Tangen, K. *Undersøkelse av hydrografiske og biologiske forhold i indre Oslofjord. Overvåkingsprogram - årsrapport 1979*. (Norwegian Institute for Water Research, 1981).

[22] Gaarder T, Gran HH (1927). Investigations of the Production of Plankton in the Oslo Fjord. Cons. Perm. Int. Pour L’Exploration La Mer Rapp. Proces-Verbaux Des Reun..

[23] Braarud T, Bursa A (1939). The Phytoplankton of the Oslo Fjord 1933–1934. Hvalrådets Skr..

[24] WoRMS Editorial Board. World Register of Marine Species, 10.14284/170 (2021)

[25] Guiry, M. D. & Guiry, G. M. AlgaeBase. World-wide electronic publication, National University of Ireland, Galway. https://www.algaebase.org; searched on 09 June 2021. (2021).

[26] Lundsør E, Stige LC, Sørensen K, Edvardsen B (2020). Long-term coastal monitoring data show nutrient-driven reduction in chlorophyll. J. Sea Res..

[27] Kraberg AC, Rodriguez N, Salewski CR (2015). Historical phytoplankton data from Helgoland Roads: Can they be linked to modern time series data?. J. Sea Res..

[28] R Core Team. R: A language and environment for statistical computing. R Foundation for Statistical Computing, Vienna, Austria. https://www.R-project.org/ (2017).

